# Development and Exploration of the Effectiveness and Feasibility of a Digital Intervention for Type 2 Diabetes Mellitus (DEsireD): Protocol for a Clinical Nonrandomized Pilot Trial in Brunei Darussalam

**DOI:** 10.2196/43208

**Published:** 2022-12-07

**Authors:** Hiu Nam Chan, Hong Shen Lim, Pui Lin Chong, Chee Kwang Yung, Musjarena Abd Mulok, Yuan Wei, Alice Moi Ling Yong

**Affiliations:** 1 EVYD Research Pte Ltd Singapore Singapore; 2 Endocrine Centre Raja Isteri Pengiran Anak Saleha Hospital Ministry of Health Bandar Seri Begawan Brunei Darussalam; 3 Department of Health Services Ministry of Health Bandar Seri Begawan Brunei Darussalam

**Keywords:** DEsireD, type 2 diabetes mellitus, digital intervention, mHealth, health coaching, chronic disease management, EMR, value-based care

## Abstract

**Background:**

The prevalence of type 2 diabetes mellitus (T2DM) is increasing worldwide. Digital interventions that incorporate the use of mobile phones and wearables have been getting popular. A combination of a digital intervention with support from professional management can enhance users’ self-efficacy better than a digital intervention alone and provide better accessibility to a lifestyle intervention. However, there are limited studies exploring the feasibility and efficacy of applying a digital intervention in Muslim-majority countries, and none have been conducted in Brunei Darussalam.

**Objective:**

The study aims to determine the effectiveness and feasibility of a proposed 16-week digital intervention program for T2DM self-management and to guide the rollout of a mobile app as part of a population health solution for adults with T2DM in Brunei. The primary outcome of this study is to measure the proportion of participants with a hemoglobin A_1c_ (HbA_1c_) reduction of at least 0.6% from baseline, and the secondary outcomes include a change in HbA_1c_, BMI, lipid profile, and EQ-5D-5L score.

**Methods:**

This single-arm nonrandomized pilot study will recruit participants using web-based (with the national health care app [BruHealth] and official social media platforms being used for outreach) and offline (in-person recruitment at health centers) approaches. A target of 180 individuals with T2DM aged between 20 and 70 years that meet the inclusion criteria will be enrolled in a 16-week digital intervention program. Baseline and postintervention markers will be evaluated.

**Results:**

The study received approval from the Medical and Health Research & Ethics Committee of the Brunei Darussalam Ministry of Health (MHREC/MOH/2022/4(1)). The recruitment process is ongoing, and we anticipate that the study will conclude by April 2023. This will be followed by data analysis and the reporting of outcomes with the intention to publish. The results of this study will be disseminated through scientific publications and conferences. This study will serve as a guide to launch T2DM digital therapeutic programs and extend to other noncommunicable diseases (NCDs) if proven as an effective and feasible approach in Brunei.

**Conclusions:**

The Development and Exploration of the Effectiveness and Feasibility of a Digital Intervention for Type 2 Diabetes Mellitus (DEsireD) study will be the first study to investigate the clinical effectiveness and feasibility of the proposed 16-week T2DM digital intervention program tailored for Brunei, a Muslim-majority country. The findings of this study can potentially scale up the proposed model of care to other NCDs as a national approach for health management solutions.

**Trial Registration:**

ClinicalTrials.gov NCT05364476; https://clinicaltrials.gov/ct2/show/NCT05364476

**International Registered Report Identifier (IRRID):**

DERR1-10.2196/43208

## Introduction

### Background

The worldwide prevalence of diabetes continues to increase, and the International Diabetes Federation (IDF) estimates the global prevalence of diabetes currently to be 9.1% with Brunei Darussalam reported to be at 12.4% among the 20-75 years old age group [1]. Type 2 diabetes mellitus (T2DM) is associated with a decreased quality of life (QOL) and increased mortality [2] and economic burden on individuals, families, and society in general [3]. Diabetes and its related complications can have devastating costs to health care systems and the national economy. According to IDF Diabetes Atlas, the global health care expenditure for the management of diabetes and diabetes-related complications was approximately US $850 billion in 2017 and is expected to increase to US $958 billion in 2045 [4].

Lifestyle interventions and modifications can effectively reduce the risk of diabetes-related complications. The American Diabetes Prevention Program [5], the Finnish Diabetes Study [6-8], and the UK DiRECT (Diabetes Remission Clinical Trial) study [9] have shown that in various populations lifestyle interventions can delay the development of T2DM and related cardiovascular complications. Lifestyle interventions remain crucial in the management of a patient with chronic disease. These interventions are traditionally performed through an in-person, face-to-face outpatient visit; such means of engagement are often plagued with challenges and often difficult to administer in the outpatient setting due to resource limitations [10,11]. The combination of offline outpatient care with a web-based software remote management model has proven to be effective in recent years [9], providing opportunities to explore further digital interventions.

Accelerated by COVID-19, digital interventions, which use different digital and mobile technologies to support health system needs, have been gaining popularity [11]. They have been shown to be safe and cost-effective in achieving positive health outcomes for T2DM [12,13]. Results from two 2020 systematic reviews and meta-analyses have shown that mobile health interventions (eg, mobile phone SMS text messages, smartphone apps, wearables, portable monitoring devices, or web-based coaching) can significantly improve hemoglobin A_1c_ (HbA_1c_) with a standardized mean difference of –0.44 [14] and a weighted mean difference (WMD) of –0.4 when compared with traditional treatment and a fasting blood glucose WMD of –0.52 [15]. A more recent systematic review in 2022 has also revealed an overall improvement in HbA_1c_ (–0.9%) compared with usual care for T2DM [16]. Digital interventions have also been used to reduce BMI and waist circumference by 1.7 kg/m^2^ and 5.77 cm, respectively [17]. On the contrary, digital interventions did not demonstrate improvement in total cholesterol, low-density lipoprotein cholesterol, or triglyceride levels [17].

Despite the proven effectiveness of digital interventions in the management of T2DM in adults, there are limited studies exploring the feasibility and efficacy of applying digital interventions in Muslim-majority countries, and no studies have been conducted in Brunei, whose population is 82.1% Muslim [18].

### Rationale of Study

This study will attempt to address the resource limitations in providing lifestyle management tools to patients with chronic diseases, specifically T2DM, by providing an adaptable and easily accessible platform to administer digital lifestyle interventions in combination with offline support.

This study is a single-arm nonrandomized clinical trial, which is the first study being conducted in Brunei to assess the potential effectiveness and feasibility of using a digital intervention for participants with T2DM. In this study, the digital intervention for participants includes the use of a mobile phone, wearable devices, and hardware to collect participants’ health information and provide telehealth consultation by health coaches based on the collected information. Through an integrated online and offline model of management, this digital intervention aims to improve diabetes management for patients in an outpatient setting. This intervention will allow accessibility to nonpharmacological lifestyle treatments outside of the hospital with the long-term goal of improving QOL and decreasing mortality, morbidity, and the economic burden of diabetes on health care resources.

### Aims and Objectives of Study

The study is conducted with the following aims: to assess the potential effectiveness and feasibility of a comprehensive digital intervention for people with T2DM, to explore the effects of a combined online and offline intervention for the management of T2DM, to improve the accessibility of a lifestyle intervention among participants with T2DM, and to understand the process of guiding the rollout of an app for T2DM management as part of a health management digital solution [19].

The primary objective is to investigate the proportion of participants with a decrease in HbA_1c_ of 0.6% through lifestyle modifications using a digital intervention after 16 weeks. Secondary objectives include estimating the change in HbA_1c_ and BMI, improvement in lipid profile components at week 16, and change in QOL measured by the EQ-5D-5L compared with baseline measurements.

## Methods

### Study Design

**Figure 1:**
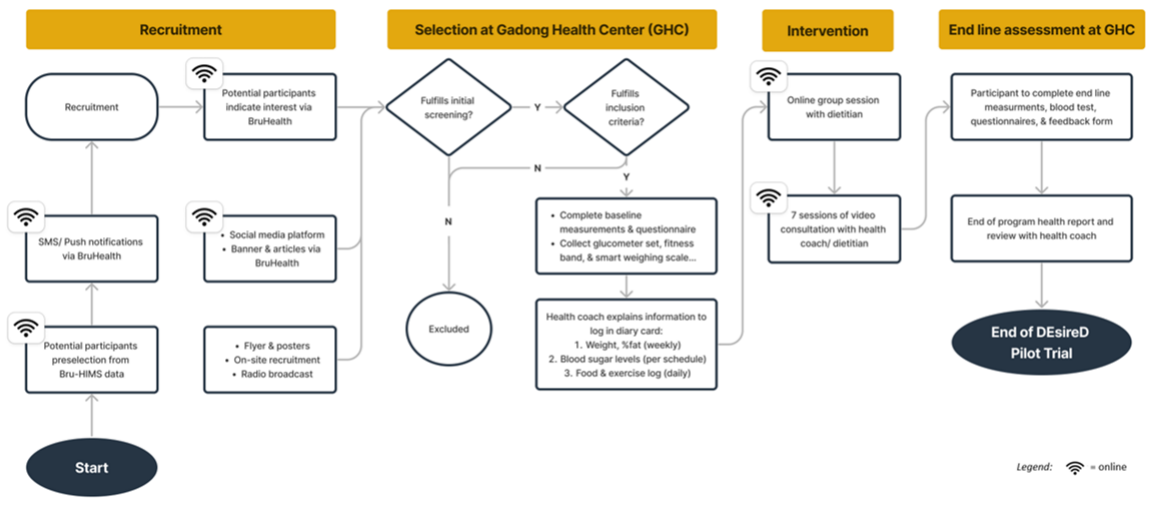
The DEsireD pilot trial study protocol. Bru-HIMS: Brunei Darussalam Healthcare Information and Management System; DEsireD: Development and Exploration of the Effectiveness and Feasibility of a Digital Intervention for Type 2 Diabetes Mellitus.

This study is a single-arm nonrandomized clinical trial that will collect participants’ baseline data, apply relevant assessment scales, and collect data using a comprehensive digital intervention in 16 weeks to evaluate the improvement of relevant markers post intervention. Figure 1 provides the standard operating procedure of the Development and Exploration of the Effectiveness and Feasibility of a Digital Intervention for Type 2 Diabetes Mellitus (DEsireD) clinical trial.

### Population

#### Inclusion Criteria

The study participants will need to meet all the following criteria: diagnosed with T2DM, HbA_1c_≥7% within 12 months prior to recruitment, age range between 20 and 70 years, and BMI between 23 and 50 kg/m^2^.

#### Exclusion Criteria

Individuals will be removed if they meet any of the following exclusion criteria: pregnant/breastfeeding; on insulin therapy or noninsulin injectable medication; a history of diabetes crisis (hypo- or hyperglycemia) in the past 6 months; blood pressure ≥160/100 mmHg; recurrent history of acute pancreatitis; decompensated liver cirrhosis; estimated glomerular filtration rate <60 ml/min/1.73 m^2^; a history of acute myocardial infarction or acute coronary syndrome (within the past 1 year), arrhythmias, or heart failure (New York Heart Association class II-IV); proliferative diabetic retinopathy; foot ulcer or gangrene; deep vein thrombosis of lower limbs (within the past 12 months); intermittent claudication; a history of cerebral hemorrhage or acute cerebral infarction (within the past 12 months); a history of active cancer; posttransplant/perioperative individuals (defined as planned for operation in the next 6 months); a history of hypo- or hyperthyroidism, including subclinical states; musculoskeletal injuries resulting in difficulty in performing physical activities; failure to provide consent; unable to perform activities of daily living; and unable to use WhatsApp and YouTube via mobile devices (eg, phone or tablet).

### Recruitment

Participants will be recruited using web-based and offline methods based on the inclusion and exclusion criteria.

#### Offline Recruitment

For offline recruitment, promotional posters and flyers will be used. These publicity materials will be placed at the front counters of health centers and in the main referral hospital (ie, the Raja Isteri Pengiran Anak Saleha [RIPAS] Hospital). Flyers will be actively given out by health coaches and recruitment teams at health centers; the flyers will be placed in the medication bags of the patients who collect oral diabetic medications from RIPAS Hospital.

#### Web-Based Recruitment

**Figure 2:**
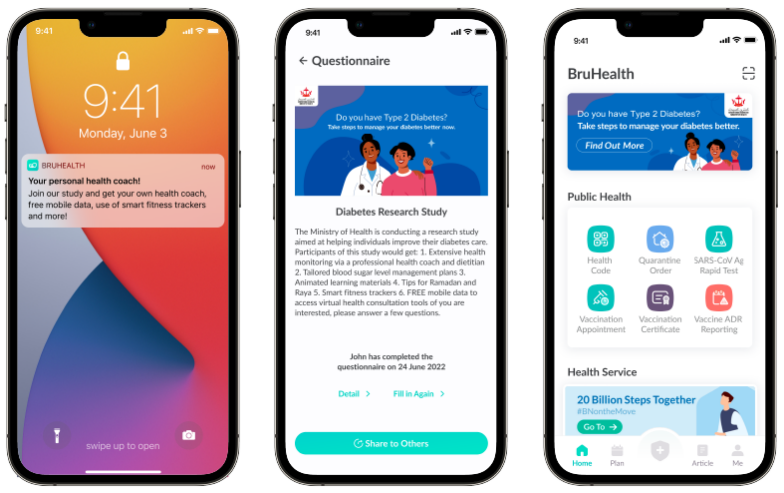
Push notification via the national health care app (BruHealth) for the Development and Exploration of the Effectiveness and Feasibility of a Digital Intervention for Type 2 Diabetes Mellitus (DEsireD) study recruitment.

In addition to traditional on-site outreach via official social media approaches, a technology-assisted adaptive recruitment strategy was used for participant recruitment [20]. Sociodemographics, comorbidities, and medication lists were extracted from the Brunei national electronic medical record system (Brunei Darussalam Healthcare Information and Management System [Bru-HIMS]) for eligibility screening. A push notification for study invitation will be sent via BruHealth to identify eligible patients with T2DM. For users who choose to response to the nudge, they will be directed to a landing page with a questionnaire for them to fill in more required details (Figure 2). While this pilot aims to recruit 180 participants, the recruitment rate was low after 3 months. Therefore, a study invitation linking to the same questionnaire was sent via SMS text message. The recruitment banner to the same link can also be seen by any BruHealth users.

A total of 9990 potential individuals with T2DM who fulfilled the inclusion and exclusion criteria (except BMI due to data unavailability) were extracted from the Bru-HIMS database, and study invitations were sent via push notifications and SMS text messages.

### Participant Selection at Baseline

Potential participants will be seen by the health coaches at the health center, where informed consent will be signed by participants prior to undergoing the screening process (see the Data Collection, Management, and Analysis: Baseline Assessment and Endline Assessment sections). The clinical investigators will review the baseline blood test results and determine whether an individual satisfies the inclusion criteria.

### Intervention

Participants will be enrolled in a 16-week program that consists of online (web-based consultations and group education sessions) and offline support (baseline and endline assessments) from health coaches and a dietitian. Participants will be given a fitness band, smart weighing scale, and glucometer set to collect one’s health information. A diet and exercise recommendation based on their submitted food and exercise log and collected health data will be provided by health coaches. A lesson plan consisting of animations, videos, infographics, and articles will be given on a weekly basis with the intention to empower participants with diabetes self-management knowledge.

The 16-week program was crafted in collaboration with the expertise of a board-certified endocrinologist in Brunei, a registered dietitian, and a physiotherapist according to evidence-based guidelines and national guidelines [21-23]. The intention is to improve the outcome of diabetes care management by influencing the participant through various methods of information via knowledge empowerment.

To ensure the successful implementation of the study and that the designed education materials are able to meet the local context, the team has worked collaboratively with diabetes specialists from the Ministry of Health, Brunei Darussalam, to design the framework of this study, oversee the program, and provide advice regarding the contents of all educational materials. To meet the cultural needs of Muslim participants, education materials that specifically focus on managing T2DM during Ramadan were developed according to relevant studies and guidelines [23-26].

### Outcome Measures

#### Primary Outcome

The primary outcome will be the proportion of participants with a decrease in HbA_1c_ by 0.6% through lifestyle modifications via digital intervention after 16 weeks.

#### Secondary Outcomes

Secondary outcomes will include a change in HbA_1c_, BMI, lipid profile, and the EQ-5D-5L components at week 16 compared with baseline readings.

### Roles and Responsibilities in the Study

#### Health Coach

The main objective of a health coach is to facilitate an efficacious and effective lifestyle intervention for the participants. The responsibilities of the health coach include providing personalized nutrition and exercise recommendations for the participants in concordance with digital management to optimize the primary and secondary outcome measures; reviewing participants’ daily self-monitoring logs of blood glucose, weight, meals, and exercise on a weekly/biweekly interval; and adhering to the escalation pathway (see Escalation Pathway section) to ensure participants’ safety.

#### Dietitian

The dietitian will lead and provide training to the team the delivery of the diabetes self-management intervention in the study with the intention to ensure the competency level of health coaches. The dietitian will also engage and empower participants’ nutrition knowledge for self-managing T2DM with web-based support via small group education sessions and web-based consultations. The dietitian is required to adhere to the escalation pathway to ensure participants’ safety. Additionally, the dietitian will assist in the evaluation of the clinical trial effectiveness and outcomes according to the collected biometric data and blood test results.

### Study Withdrawal

The participant may withdraw from the trial at any time by withdrawing their informed consent. An end-of-study form will be given, and the reason for withdrawal will be documented.

### Statistical Analysis: Sample Size Calculation

A statistical power calculation was performed as follows: a sample size of 120 achieves 80% power to detect a proportion of 0.63 using a 1-sided exact test with a significance level of 0.025. These results assume that the proportion of the population under the null hypothesis is 0.5. Considering a dropout rate of 30%, the target sample size is 180.

### Data Collection, Management, and Analysis

#### Baseline Assessment

During the screening process at baseline, participants will undergo a panel of blood tests to determine eligibility for study participation. Baseline blood tests include full blood count, HbA_1c_, fasting blood glucose, fasting lipid profile, liver function test, renal panel, and thyroid function test. The clinical investigators will review baseline blood test results and determine whether potential participants satisfy the inclusion criteria. Eligible participants will be enrolled in the study, asked to complete the EQ-5D-5L questionnaire, provided with loaned devices, and requested to submit data required on a weekly basis at enrollment.

#### Study Assessment

During the 16-week study period, participants will be required to log their blood glucose levels, weight, and heart rate using the loaned devices into a daily report card. They will also be required to manually record their meals and exercise in the same report card. Reviews of logs will be performed on a weekly interval by the health coaches.

#### Endline Assessment

Upon completion of the 16-week intervention, participants will undergo a second panel of blood tests, which include full blood count, HbA_1c_, fasting blood glucose, fasting lipid profile, and liver function test as part of the primary and secondary outcome measures. Participants will also be asked to complete the EQ-5D-5L questionnaire to assess participants’ QOL in comparison to baseline.

#### Data Management

A dedicated proprietary research platform called EVYDResearch (EVYD Technology Sdn Bhd) will be used for data management. Data confidentiality will be maintained through EVYDResearch, as only the research team will have access to the full information. Sociodemographic and clinical variables will be extracted from Bru-HIMS and integrated into EVYDResearch. Data collected via questionnaires and diaries will be manually transcribed and housed in EVYDResearch. Physical copies of all the collected data, such as case report forms, informed consent forms, and questionnaires, will be locked in a cabinet that is only accessible to the research team. All data will be stored for 5 years and destroyed thereafter in compliance with the Ministry of Health, Brunei Darussalam regulations.

#### Statistical Analysis

Descriptive statistics will be performed for both primary and secondary outcomes. Frequency and count will be calculated for categorical variables. Means, SDs, and quartiles will be calculated for continuous variables. EVYDResearch will be used for simple data validation rules, such as uniqueness and missingness; descriptive statistical analysis for primary and secondary outcomes; and simple visualization plots. Additionally, R (R Foundation for Statistical Computing) or other software may be used when necessary to perform more advanced statistical analysis and data visualization.

### Event Safety Monitoring

The terms pertaining to events have been adapted from ClinicalTrials.gov [27] and are as follows:

Adverse Events are unfavourable changes in health, including abnormal laboratory findings, that occur in trial participants during the clinical trial. Serious Adverse Events include adverse events that result in death or result in either inpatient hospitalization or the prolongation of hospitalization, are life-threatening or result in a persistent or significant disability/incapacity. Other important medical events, based on appropriate medical judgment, may also be considered Serious Adverse Events if a trial participant's health is at risk and intervention is required to prevent an outcome mentioned. Other Adverse Events are adverse events that are not Serious Adverse Events but exceed the indicated frequency threshold.

All adverse events will be logged in the adverse events form. Any serious adverse events (expected/unexpected) will be reported to the Brunei Darussalam Medical and Health Research & Ethics Committee (MHREC) by the principal investigator within 24 hours of the event through the adverse events form.

For medical events, health coaches will review the weekly report card and follow the escalation pathway as described below.

### Escalation Pathway

#### Health Coach to Dietitian

Participants with poorly controlled capillary blood glucose (defined as blood glucose <4 mmol/L and >16.6 mmol/L) will be referred for web-based counseling by the dietitian.

#### Dietitian to Clinical Investigators

Participants with any of the following will be referred to the clinical investigators: two episodes of hypoglycemia, which is defined as a blood glucose of <4.0 mmol/L with or without symptoms of hypoglycemia, despite diet and lifestyle modifications, and more than 2 occasions of hyperglycemia, which is defined as a blood glucose of >16.6 mmol/L in a week.

#### Clinical Investigators to Diabetes Nurse Educators

In the respective clinic where participants see their primary physician for diabetes care, participants who require counseling about the timing of medications to meals and exercise will be referred to the diabetes nurse educators.

#### Consultations With Clinical Investigators

**Figure 3:**
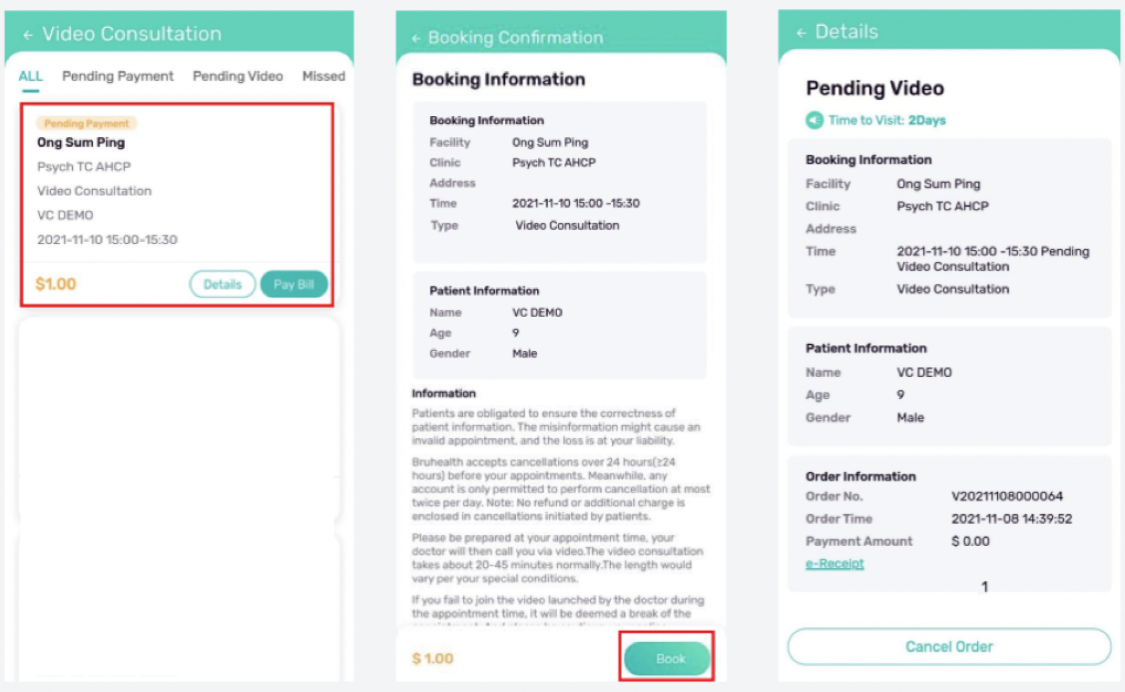
Video consultation appointment arranged via BruHealth app.

**Figure 4:**
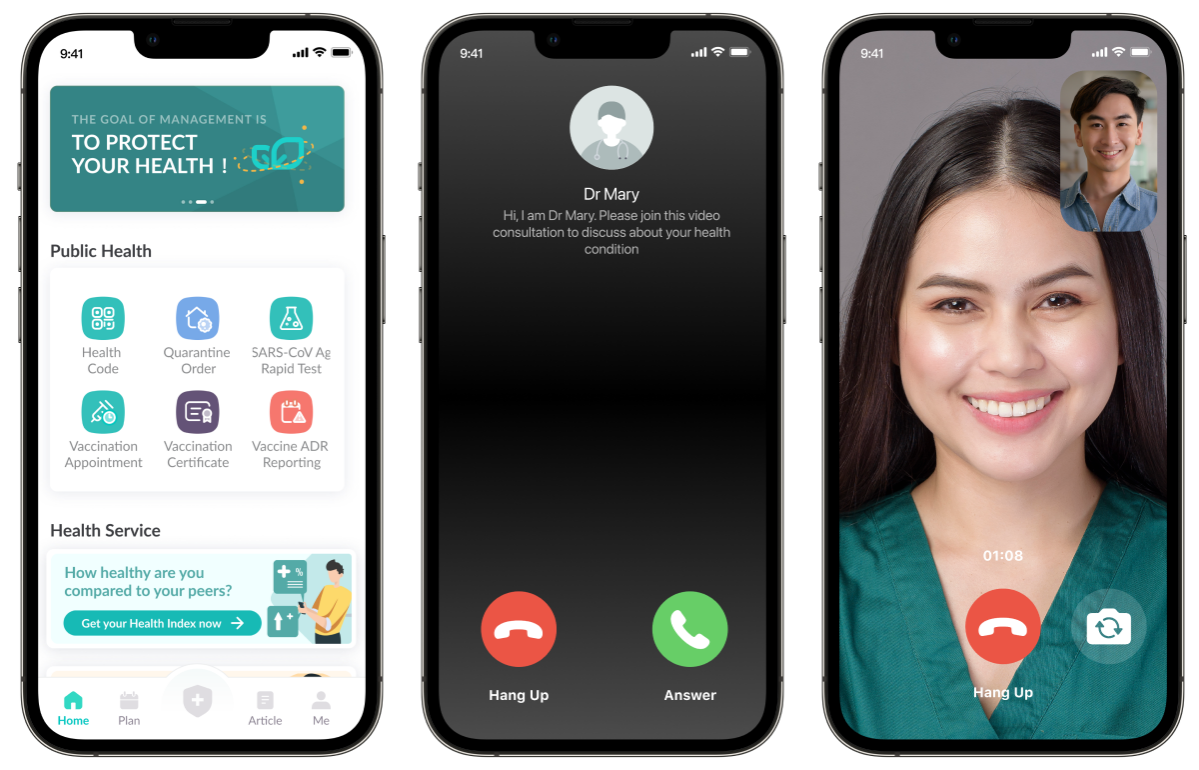
Video consultation interphase via the national health care app (BruHealth).

Consultations with clinical investigators will only be done via the virtual clinics (refer to Figures 3 and 4) that are specifically set up for this study period. Participants with recurrent hypoglycemia and hyperglycemia despite lifestyle modification will be excluded from the study for the participant’s safety. The clinical investigators will write a letter to inform the participant’s primary physician for an urgent review of their treatment. However, participants who do not experience any of the above issues will continue their routine clinic visits (if any) with their primary physician during the study period, and any changes in medications will be noted. Such changes in medication are allowed as part of participants’ usual care and do not constitute an exclusion from the study.

### Ethics Approval

This study is being conducted in accordance with Good Clinical Practice, as defined by The International Council for Harmonisation. The trial protocol has received approval from MHREC from the Ministry of Health Brunei (MHREC/MOH/2022/4(1)). The appropriate participant information sheet and informed consent form describing in detail the trial interventions, trial procedures, and risks were approved by the ethical committees. Aside from providing a participant information sheet to all potential participants, the investigator will explain the study and answer any questions posed. After being given adequate time to consider the information, the participant will be asked to sign the informed consent document. The participant may withdraw from the trial at any time by withdrawing their informed consent. The rights and welfare of the participants will be protected by emphasizing to them that the quality of medical care will not be adversely affected if they decline participation.

## Results

The enrollment period of this study is between April 18 and November 30, 2022. As of November 4, 2022, a total of 72 participants have been recruited. In this study, participants are recruited in batches, and at the data collection level, there are 12 participants who have completed the endline assessment. Data collection is ongoing. We anticipate this study to conclude by April 2023, followed by data analysis and final reporting with the intention to publish.

## Discussion

### Overview

This study protocol provides an overview of the methodology used in the DEsireD clinical trial. To our knowledge, this study is the first to examine the effectiveness of a proposed 16-week digital intervention program for T2DM self-management and to explore the feasibility of the proposed model of care—a combination of online and offline management to improve the accessibility of a lifestyle intervention among individuals with T2DM in Brunei.

With resource limitations in providing holistic lifestyle interventions in a conventional manner through face-to-face consultations in outpatient settings, digital interventions can be a scalable and cost-effective solution for diabetes self-management. According to Koh et al [19], COVID-19 has had a negative impact on noncommunicable diseases (NCDs) in Brunei due to the disruption of essential health services. Thus, the government has stated its intention to use BruHealth to prioritize NCD service expansions. This includes a diabetes digital intervention. Globally, there are multiple studies demonstrating the effectiveness of digital interventions [12-17,28,29], but there are limited studies conducted in Southeast Asia, particularly in Muslim-dominant populations [16]. This study will be useful for health care professionals and policy makers to understand the possible barriers to the implementation process during the subsequent phases of rolling out an app for T2DM management as a nationwide digital health solution in Brunei [19]. In addition, a feedback form will be given to any participants who have dropped out or completed the study to collect their quantitative feedback on the DEsireD trial. This will include overall experience, the usefulness of the designed program to assist them with setting goals, study duration (16 weeks), and qualitative feedback on the overall perception and contextual feasibility.

The majority of studies use the reduction of the HbA_1c_ level as a clinical marker for the effectiveness of digital interventions for patients with T2DM [16,30]. We agree with Stevens et al [16] that a wider measure of clinical effectiveness should go beyond HbA_1c_. Thus, this study will investigate changes in fasting blood glucose, lipid profile, and health-related QOL (measured by the assessment of the EQ-5D-5L [31,32]).

There are several limitations to this study. The first is achieving the targeted sample size. This will make the evaluation of the primary objective difficult. There is a sample of convenience, which may result in response bias. The nature of a single-arm study will be limited by its nonrandomized design of unknown causal inference. Finally, in the absence of comparison, the reference end point of a 0.6 reduction in HbA_1c_ over 16 weeks was used as a benchmark for clinical effectiveness. Our rationales include the effect of other mobile health interventions on the reduction of HbA_1c_ levels are between a WMD of 0.40 to a mean average of 0.9 [15,16] and the effect of oral diabetic medication as monotherapy is expected to reduce HbA_1c_ by 0.5 to 2.0 [33]. Thus, a lower average number of 0.6 was used. In addition, the absolute and relative decrease in HbA_1c_ will be calculated as secondary outcomes.

This study is expected to conclude in April 2023. The scientific findings will be presented as oral communications and abstracts at regional, national, and international scientific meetings related to T2DM. The findings will also be published in peer-reviewed journals.

### Conclusions

The DEsireD study will be the first study to investigate the clinical effectiveness of the proposed 16-week digital intervention program tailored for individuals with T2DM in Brunei.

The evidence will serve as a guide to roll out a nationwide value-based care program via an app as a digital solution. The lessons from the implementation of this study and the feedback and data from participants will be beneficial for further localization of this digital intervention program. Consequently, scaling up to other NCDs and validation with a larger sample size to demonstrate that the digital intervention is a safe, scalable, sustainable, and cost-effective approach in Brunei will be needed.

This research received no specific grant from any funding agency in the public, commercial, or not-for-profit sectors.

## Abbreviations

Bru-HIMS: Brunei Darussalam Healthcare Information and Management System
DEsireD: Development and Exploration of the Effectiveness and Feasibility of a Digital Intervention for Type 2 Diabetes Mellitus
DiRECT: Diabetes Remission Clinical Trial
HbA_1c_: hemoglobin A_1c_
IDF: International Diabetes Federation
MHREC: Medical and Health Research & Ethics Committee
NCD: noncommunicable disease
QOL: quality of life
RIPAS: Raja Isteri Pengiran Anak Saleh
T2DM: type 2 diabetes mellitus
WMD: weighted mean difference

## Data Availability

The data collection process is still underway. The other materials presented in the study are included in the paper. Further inquiries can be directed to the corresponding author.
